# Novel sandwich immunoassay detects a shrimp AHPND-causing binary PirAB^Vp^ toxin produced by *Vibrio parahaemolyticus*

**DOI:** 10.3389/fcimb.2023.1294801

**Published:** 2023-11-27

**Authors:** Min-Young Jeon, Jee Eun Han, Dong Gwang Lee, Young-Lai Cho, Ju-Hong Jang, Jangwook Lee, Jong-Gil Park, Do Hyung Kwon, Seon Young Park, Wantae Kim, Kyunglee Lee, Ji Hyung Kim, Nam-Kyung Lee

**Affiliations:** ^1^ Biotherapeutics Translational Research Center, Korea Research Institute of Bioscience & Biotechnology (KRIBB), Daejeon, Republic of Korea; ^2^ Department of Biochemistry, College of Natural Sciences, Chungnam National University, Daejeon, Republic of Korea; ^3^ Laboratory of Aquatic Biomedicine, College of Veterinary Medicine, Kyungpook National University, Daegu, Republic of Korea; ^4^ Environmental Diseases Research Center, Korea Research Institute of Bioscience and Biotechnology, Daejeon, Republic of Korea; ^5^ Division of Animal and Dairy Sciences, College of Agriculture and Life Science, Chungnam National University, Daejeon, Republic of Korea; ^6^ Cetacean Research Institute, National Institute of Fisheries Science, Ulsan, Republic of Korea; ^7^ Department of Food Science and Biotechnology, College of BioNano Technology, Gachon University, Seongnam, Republic of Korea

**Keywords:** vibrio parahaemolyticus, PirAB^Vp^ toxin, acute hepato-pancreatic necrosis disease, shrimp, sandwich immunoassay

## Abstract

**Introduction:**

The binary PirA/PirB toxin expressed by *Vibrio parahaemolyticus* (PirAB^Vp^) is a virulent complex that causes acute hepatopancreatic necrosis disease (AHPND) in shrimps, affecting the global shrimp farming industry. AHPND is currently diagnosed by detecting *pirA* and *pirB* genes by PCR; however, several *V. parahaemolyticus* strains do not produce the two toxins as proteins. Thus, an immunoassay using antibodies may be the most effective tool for detecting toxin molecules. In this study, we report a sandwich ELISA-based immunoassay for the detection of PirAB^Vp^.

**Methods:**

We utilized a single-chain variable fragment (scFv) antibody library to select scFvs against the PirA or PirB subunits. Phage display panning rounds were conducted to screen and identify scFv antibodies directed against each recombinant toxin subunit. Selected scFvs were converted into IgGs to develop a sandwich immunoassay to detect recombinant and bacterial PirAB^Vp^.

**Results:**

Antibodies produced as IgG forms showed sub-nanomolar to nanomolar affinities (K_D_), and a pair of anti-PirA antibody as a capture and anti-PirB antibody as a detector showed a limit of detection of 201.7 ng/mL for recombinant PirAB^Vp^. The developed immunoassay detected PirAB^Vp^ in the protein lysates of AHPND-causing *V. parahaemolyticus (Vp^AHPND^)* and showed a significant detectability in moribund or dead shrimp infected with a *Vp^AHPND^* virulent strain compared to that in non-infected shrimp.

**Discussion:**

These results indicate that the developed immunoassay is a reliable method for diagnosing AHPND by detecting PirAB^Vp^ at the protein level and could be further utilized to accurately determine the virulence of extant or newly identified *Vp^AHPND^* in the global shrimp culture industry.

## 1 Introduction

Acute hepatopancreatic necrosis disease (AHPND or early mortality syndrome) is a bacterial disease that causes significant economic losses to the global shrimp aquaculture industry (Shinn et al., 2015). Since its first outbreak in China in 2009, AHPND has been reported in several countries of the Americas and Asia (Global Seafood Alliance, 2012; Tran et al., 2013; Joshi et al., 2014; de la Peña et al., 2015; Soto-Rodriguez et al., 2015; Tun et al., 2017; Eshik et al., 2018; Dhar et al., 2019). Moreover, AHPND was reported in Korea in 2016, causing serious economic losses (Han et al., 2020). The causative agent of AHPND has been confirmed to be *Vibrio parahaemolyticus* strains (*Vp^AHPND^*), which express a binary PirAB^Vp^ toxin homologous to the *Photorhabdus* insect-related toxin (Han et al., 2015; Lee et al., 2015; Sirikharin et al., 2015). The production of the PirAB^Vp^ toxin in *Vp^AHPND^* is responsible for its cytotoxic effect on the epithelial cells of the shrimp hepatopancreas (HP) in the presence of a large (~70 kb) conjugative plasmid (pVA1) that contains the two toxin-subunit genes (*pirA* and *pirB*) (Yang et al., 2014; Han et al., 2015). Although the exact functions of the PirA and PirB subunits are still uncertain, the PirA subunit may play an initial stabilizing role, allowing PirB to bind with higher affinity to different glycan receptors located on the surface of the HP (Soto-Rodriguez et al., 2022).

Therefore, several DNA-based detection methods targeting toxin genes (*pirA* and *pirB*) and plasmid pVA1 have been developed, as recommended by the OIE (World Organization for Animal Health, 2017). However, those diagnostic techniques have faced difficulties due to the peculiar characteristics of the AHPND-causing pathogens as follows: (i) the emergence of mutant *Vp^AHPND^* strains which have deletions of entire or partial *pirA* and/or *pirB* genes has been reported (Han et al., 2016); (ii) the emergence of atypical *Vp^AHPND^* strains which contain full-length *pirA* and *pirB* but do not produce PirA^Vp^ toxins and fail to cause AHPND has been reported (Vicente et al., 2019). Therefore, there is an urgent need to develop a protein-based assay to detect the PirA and PirB subunits to provide more accurate and valid PCR-based assays for the detection of AHPND-causing pathogens.

Studies have reported that antibody-based immunoassays can be used to detect toxic subunits at the protein level. Monoclonal antibodies (mAbs) were previously developed by immunizing mice with concentrated culture supernatants prepared from *Vp^AHPND^* isolates, and PirA or PirB subunits were detected by dot and western blotting analyses (Wangman et al., 2017; Wangman et al., 2018). In a recent study, an indirect enzyme-linked immunosorbent assay (iELISA) was developed with mAbs, which were generated by immunization with recombinant PirA or PirB; the indirect ELISA method detected each toxin subunit in protein lysates from several bacterial strains and AHPND-challenged shrimps (Mai et al., 2020). These antibody-based assays were successful in detecting either PirA or PirB subunit proteins; however, it has been shown that PirA and PirB interact with each other to form a heterodimeric PirAB^Vp^ and the complex causes significant mortality and morbidity in shrimp (Lee et al., 2015). These findings indicate that the virulence of AHPND-causing *Vibrio* spp. depends on heterodimeric PirAB^Vp^. Thus, detecting the heterodimeric toxin complex is a challenging task for diagnosing AHPND in practice.

To the best of our knowledge, no antibody-based immunoassays are available for the detection of the heterodimeric protein complex PirAB^Vp^. In this study, we developed a novel sandwich immunoassay to detect toxin complexes. Recombinant PirA and PirB subunits were produced and used as antigens for phage display panning using a naïve human single-chain variable fragment (scFv) antibody library. Selected scFvs were converted to IgG forms, and anti-PirA and anti-PirB antibodies were utilized to develop a sandwich ELISA-based immunoassay for capture and detection, respectively. The immunoassay exhibited specific detection activity for heterodimeric PirAB^Vp^, but not for PirA or PirB, and successfully detected heterodimeric PirAB^Vp^ in protein lysates extracted from several Vibrio spp. and *Vp^AHPND^*-infected shrimp.

## 2 Materials and methods

### 2.1 Cloning and production of recombinant PirA and PirB subunits

Full-length protein-coding sequences of *pirA* and *pirB* were obtained by PCR using the Korean *Vp* ^AHPND^ strain 19–021-D1 as a DNA template (Han et al., 2020). PCR amplifications were conducted using the primer sets Vp_PirA_1F/Vp_PirA_336R and PirB_1F/PirB_1317R, according to Lee et al. (2015). After the exact nucleotide sequences were confirmed, the codons of *pirA* and *pirB* genes were optimized for expression in the *Escherichia coli* BL21 (DE3) expression system using the algorithm “OptimumGene” (GenScript, USA), synthesized *de novo*, and cloned into the pET-21b (+) expression vector (Novagen, USA). Recombinant protein expression and purification services were provided by Customs Protein Purification Service (AbClon Inc., Seoul, Korea).

### 2.2 Selection and screening of anti-PirA and anti-PirB scFv antibodies

For antibody selection, a naïve phage antibody display library from pooled healthy donors was constructed using the pADL-22c phagemid vector (Antibody Design Labs, USA), as described previously (Lee et al., 2018). Recombinant PirA or PirB was prepared at 10 μg/mL in phosphate buffer saline (PBS, pH 7.4) and coated on immunotube (Thermo Scientific, USA) by overnight incubation at 4°C. Immunotubes coated with each toxin subunit were incubated with phages that had been pre-blocked in PBS/2% nonfat milk (MPBS) for 1 h. Immunotubes were then washed with PBS/0.05% Tween20 (PBST) and the bound phages were eluted and propagated as previously described (Lee et al., 2018). To assess the binding activity of polyclonal scFvs, 10 μl of outputs from each round was inoculated in 5 ml 2xYT/Ampicillin (100 μg/ml) supplemented with 2% glucose and cultured until the OD600 reaches 0.4–0.5. Bacteria was pelleted by centrifugation and resuspended using 5 ml 2xYT/Ampicillin supplemented with 1 mM isopropyl β-D-1-thiogalactopyranoside (IPTG), then cultured at 30°C with shaking for 16 hr. The culture was centrifuged at 4,000 rpm for 15 min and lysed with 125 μl of periplasmic extraction buffer (PEB; 30 mM Tris-HCl/20% sucrose/1 mM EDTA, pH 8.0), followed by additional periplasmic extraction using 250 μl of 5 mM MgSO_4_. Two supernatant lysates extracted using PEB and MgSO_4_ were mixed and used for ELISA with HRP-conjugated anti-HA antibody (Roche, Switzerland) as a secondary antibody. To screen individual scFv antibodies, single colonies picked from the output of 2^nd^ and 3^rd^ rounds were inoculated into 150 μl of 2xYT/Ampicillin/2% glucose in a 96-well U-bottom culture plate and grown for 3 hr. Bacteria was pelleted by centrifugation, resuspended with 150 μl of 2xYT/Ampicillin with 1 mM, and grown for overnight at 30°C with shaking and proper aeration. After IPTG induction, the plate was centrifuged and scFvs extracted from each pellet using 100 μl of PEB were subjected to screening by ELISA using HRP-conjugated anti-HA antibody. Clones that demonstrated at least five-fold binding over background were submitted for Sanger sequencing analysis (Bioneer, Republic of Korea) using a pelB-Forward primer (5′-AATACCTATTGCCTACGGCTG-3′) for identifying the variable heavy (VH) or variable light (VL) region sequence.

### 2.3 scFv purification

Selected monoclonal scFvs were cultured in 100 ml 2xYT/Ampicillin/2% glucose until the absorbance at 600 nm was between 0.5–0.8. bacterial culture was centrifuged, and the pellet was resuspended in 100 ml of 2xYT/Ampicillin/1 mM IPTG and grown for overnight at 30°C with vigorous shaking. The periplasmic fraction was obtained using 2.5 ml of PEB, followed by resuspension of the remaining pellet in 5 ml of 5 mM MgSO_4_. The two fractions from the extraction procedure were mixed and subjected to scFv purification using an NGC Quest 100 column (Bio-Rad Laboratories, USA) equipped with a HisTrap™ HP column (Cytiva, USA).

### 2.4 Reformatting, transient expression, and purification of IgGs

Using pcDNA3.4 as a backbone vector, heavy chain (HC) and light chain (LC) expression vectors were constructed by inserting HC (CH1–CH2–CH3) and LC (CL) constant regions, respectively. Restriction enzyme sites were inserted in each expression vector for cassette cloning of VH and VL. The VH or VL genes in each scFv were amplified by PCR with the appropriate restriction enzyme sites and cloned into the HC or LC expression vector. HC and LC expression vectors for producing each IgG were co-transfected into Expi293F cells (1 × 10^8^) in 50 ml Expi293F expression medium. Cells were cultured for 5 days post-transfection, and supernatants were harvested by centrifugation at 4,000 rpm for 40 min and filtered using a 0.22-μm bottle-top vacuum filter. The produced IgGs were purified using an NGC Quest 100 equipped with a MabSelect™ PrismA column (Cytiva, USA), dialyzed in PBS (pH 7.4), and analyzed by SDS-PAGE.

### 2.5 Indirect ELISA

To validate the binding of purified scFvs or IgGs by ELISA, recombinant PirA or PirB diluted in 100 μl of PBS (200 ng/well) was coated on a 96-well plate for overnight. The plates were washed with PBST and blocked with MPBS at room temperature for 2 h. Various concentrations of the antibodies were allowed to bind to each antigen for 1 h, followed by three washes with PBST. Bound scFvs and IgGs were detected using horseradish peroxidase (HRP)-conjugated anti-HA antibody and goat anti-human IgG (Fc-specific) (Thermo Scientific), respectively, by reacting for 30 min at room temperature. After washing with PBST three times, 100 μl of OptEIA tetramethylbenzidine (TMB) (BD Biosciences, USA) was added to each well and incubated for 5 min, followed by terminating the reaction using 100 μl of 2N H_2_SO_4_. Absorbance was measured at 450 nm using a SpectraMax ABS Plus plate reader (Molecular Devices, USA).

### 2.6 BLI Analysis

An amine-reactive 2^nd^ generation (AR2G) biosensor (ForteBio, USA) was used to immobilize recombinant PirA or PirB as described previously (Jang et al., 2022). The biosensors were quenched with 1 M ethanolamine–HCl (pH 8.5) for 300 s, and a zero baseline was obtained with PBS for 120 s. Kinetic analysis was performed using the Octet K2 system (ForteBio) by determining the association (K_on_) and dissociation (K_off_) of anti-PirA and anti-PirB antibodies for 600 s. The K_D_ values of each antibody were calculated based on K_on_ and K_off_ using data analysis software (HT 12.0; ForteBio).

### 2.7 Detection of PirA or PirB from AHPND-associated *Vibrio* strains

Four AHPND-pathogenic strains of *V. parahaemolyticus* (19-022-A1, 19-021-01, CH49, 13-028-A3), one AHPND-mutant strains of *V. parahaemolyticus* (13-511-A2), and one *V. harveyi* strain (LB4) originated from different geographical origins were used in this study (8, 10, 20) (**Table 1**). *Vibrio* spp. were isolated from the stomachs of moribund shrimp and bacterial identifications were carried out by PCR assay targeting species specific genes (toxR and TopA genes), and PCR assay targeting *pirA*- and *pirB*-like genes (Han et al., 2015). These strains were overnight cultured in TSB + (Tryptic soy broth plus 2% NaCl) at 28–29°C with gentle (100 rpm) shaking and cell pellets were obtained by centrifugation at 8,000 x g for 15 min. To extract total proteins, the pellets were lysed using 4 ml B-PER^®^ bacterial protein extraction reagent (Thermo Fisher Scientific, USA) supplemented with 27.3 U of DNase (Zymo Research, USA) and EDTA-free protease inhibitor (GenDEPOT, USA) in ice for 20 min, and protein lysates were harvested by centrifugation at 15,000 × g for 5 min. Lysates (250 μl/well) were coated on a 96-well plate and PirA or PirB was detected by ELISA, as described in the *Indirect ELISA* section.

**Table 1:** Measurement of antibody affinity based on biolayer interferometry.

mAb	K_D_ (M)	K_on_ (1/Ms)	K_off_ (1/s)	R^2^
1A8	1.675E-09	1.841E05	3.084E-04	0.987
3A5	2.883E-10	7.532E05	2.171E-04	0.9812

### 2.8 Sandwich ELISA

100 μl of anti-PirA IgG (15 μg/ml) was coated on a 96-well plate for overnight at 4°C. After blocking with MPBS for 1 h, a mixture of recombinant PirA and PirB at 1:1 ratio (each 100 nM) was serially diluted in PBS and added to each well, followed by incubation for 2 h at room temperature. Anti-PirB IgG was biotinylated for detection as previously described (Jang et al., 2022). After washing the plate with PBST three times, 100 μl of biotinylated anti-PirB IgG (15 μg/ml) was added to each well and incubated for 1 hr at room temperature. The plate was washed thrice with PBST and incubated with HRP-conjugated streptavidin (Sigma Aldrich, USA) for 1 h at room temperature. After washing thrice with PBST, a colorimetric reaction was conducted as described in the *Indirect ELISA* section.

### 2.9 Detection of PirAB^Vp^ in hepatopancreas lysates of *Vp^AHPND^*-infected shrimps

17 and 15 P*. vannamei* (mean weight = 1 g) were stocked in 17-L tanks as infected and non-infected groups. *Vp^AHPND^*-causing strain 13-028/A3 was grown to 1 × 10^9^ CFU/ml, mixed with shrimp feed at a 1:1 ratio, and fed to the infected group. In the non-infected group, the shrimp were fed normally without bacteria. On day 7, all shrimp were dissected under aseptic conditions to prepare HP, as described previously (Han et al., 2020). The HP samples were kept at –80°C until further use. The animal use and experimental protocols were reviewed and approved by the Animal Research Ethics Committee of the Korea Research Institute of Bioscience and Biotechnology (IACUC approval no. KRIBB-AEC-21119; April 2021). To extract total protein from shrimp HP, the stored samples were thawed at 4°C and lysed using 300 μl of 1X RIPA lysis buffer (Merck Millipore, USA) supplemented with phosphatase and protease inhibitors (GenDEPOT) in ice for 40 min. Total protein lysates were obtained by centrifugation at 10,000 rpm for 40 min, and the protein concentration was measured using the BCA assay. Protein lysates (20 μg/well) were coated and reacted to detect PirA or PirB as described in the *Indirect ELISA* section and PirAB^Vp^ as described in the *Sandwich ELISA* section.

### 2.10 Theoretical limit of detection

LoD was defined as the lowest PirAB^Vp^ concentration of the detected colorimetric signal, which was greater than non-specific binding. Various concentrations of recombinant PirAB^Vp^ or protein lysates were analyzed by sandwich ELISA, and linear regression analyses were conducted using GraphPad Prism 8.0 (GraphPad Software, USA). The standard deviation of the response (σ) and the slope (S) of the linear regression curve were used to calculate LoD using the equation described below.


LoD =3.3 × σ/S

### 2.11 Antibody modeling and docking analysis

The structural model of the antibody variable fragment (Fv) was generated using RosettaAntibody on the ROSIE server using the VH and VL chain sequences of each antibody, as described previously (Weitzner et al., 2017). Docking models between 1A8 Fv and PirA (PDB:3X0T) or between 3A5 and PirB (PDB:3X0U) were generated using ZDOCK (Pierce et al., 2014). Fv-binding amino acid residues on either PirA or PirB (≤ 4 Å) were analyzed from each docking model and visualized using the PyMOL Molecular Graphics System.

### 2.12 Statistical analysis

The data are shown as the means ± the standard deviation (SD) of the means, and statistical data analyses were performed via unpaired two-tailed Student’s t-tests using GraphPad Prism 8.0 (GraphPad Software, USA). *P* values of < 0.05 indicated statistically significant differences.

## 3 Results

### 3.1 Screening of scFv antibodies binding to PirA or PirB subunit

**Figure 1:**
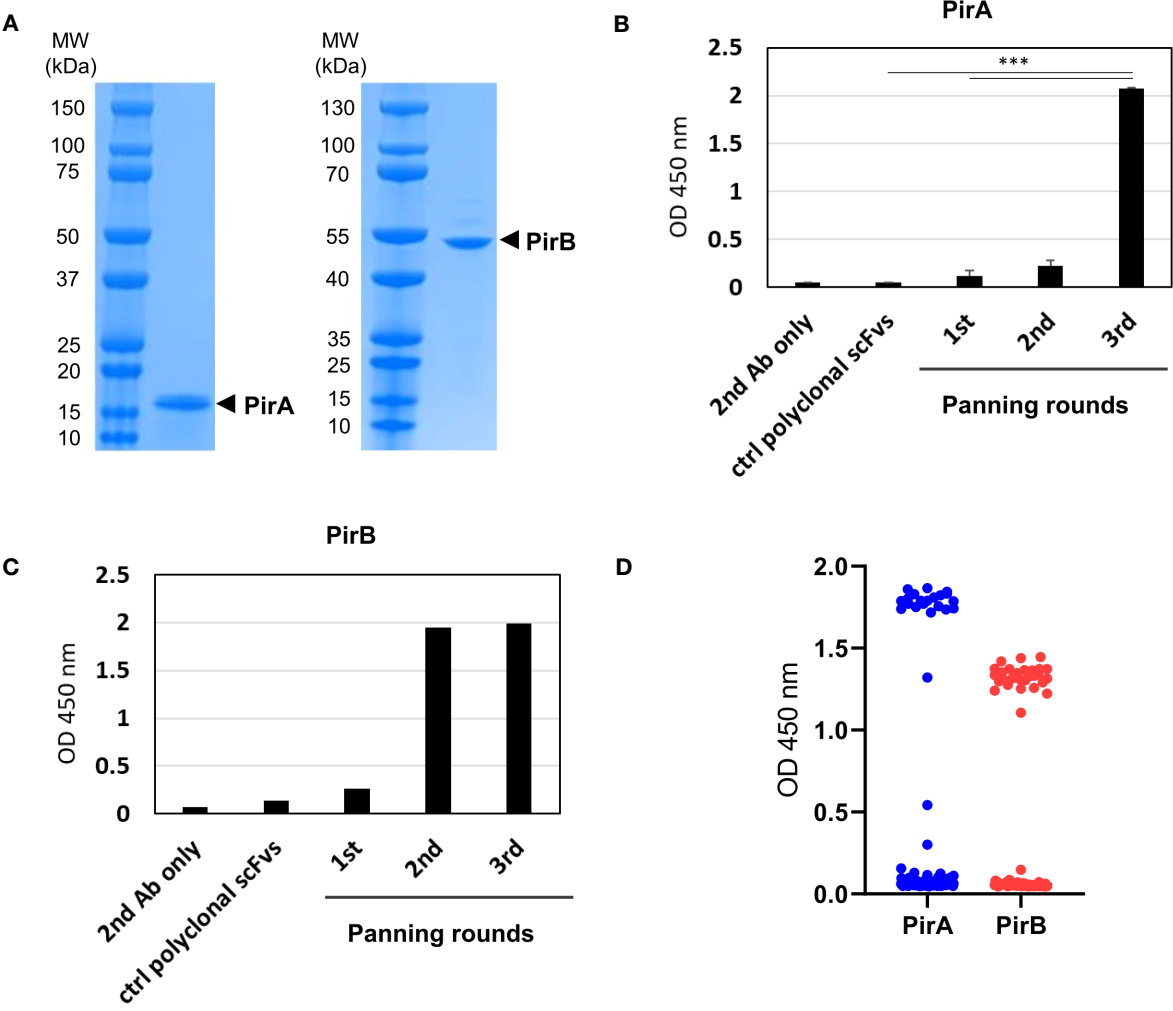
Phage display panning and screening of scFv antibodies binding to recombinant PirA or PirB. **(A)** Production of recombinant PirA and PirB proteins. E. coli BL21-DE3 transformed with PirA- or PirB-expressing vector was cultured and induced by IPTG for protein expression. Each toxin subunit expressed with a 6xHis tag was purified by Ni-NTA affinity chromatography, and the molecular weight of PirA (~13 kDa) and PirB (~50 kDa) was validated using SDS-PAGE. **(B, C)** Phage display panning using PirA and PirB. A naïve human scFv phage library was allowed to bind to each toxin subunit coated on an immunotube, then panning rounds were performed by following washing and elution procedure. After 3rd round of panning, polyclonal scFv antibodies from each round were expressed by IPTG induction and used to evaluate their binding activity to **(B)** PirA, or **(C)** PirB, by ELISA. Data represent the mean ± SD of duplicate tests **(B)**. ***P < 0.001. **(D)** Screening of anti-PirA and anti-PirB scFv antibodies. Individual TG1 colonies from the 2^nd^ and 3^rd^ round outputs were inoculated and cultured to express scFv antibodies in periplasmic region by IPTG induction. Screening of scFvs obtained from periplasmic fraction was conducted on an ELISA plate coated with each antigen. The positive ratio (OD 450 nm > 0.5) of clones tested was approximately 15% (22/144) for PirA and 31% (30/96) for PirB, respectively.

To prepare recombinant PirA and PirB, plasmids encoding each toxin gene with a 6xHis tag were separately transformed into E. coli BL21 (DE3) cells, and the recombinant toxins were expressed by IPTG induction. Pure recombinant PirA or PirB proteins corresponding to approximately 13 or 50 kDa were purified using a Ni-NTA column and analyzed by SDS-PAGE and Coomassie Brilliant Blue staining (**Figure 1A**). To select scFv antibodies against the toxins, we utilized a naïve human scFv antibody library and performed three iterative panning rounds using purified recombinant PirA or PirB. After the 3^rd^ round of panning, polyclonal scFv antibodies from each round were expressed by IPTG induction and their binding activity to each antigen was evaluated using ELISA. As shown in **Figure 1B**, the binding of polyclonal anti-PirA scFv antibodies expressed from the 3^rd^ round output was highly increased compared to that of polyclonal scFv antibodies derived from the previous rounds or the original library, indicating that the panning rounds were successfully performed and target-binding clones were amplified in the 3^rd^ round output. In addition, polyclonal scFvs expressed in the 2^nd^ and 3^rd^ rounds showed similarly increased binding to PirB (**Figure 1C**). By screening individual scFv clones from the 3^rd^ round of output, we found 22 and 30 positive binders to PirA and PirB, respectively (**Figure 1D**), and performed DNA sequencing analysis of the clones to determine the sequences of the variable heavy (VH) and variable light (VL) chains.

### 3.2 Evaluation of selected anti-PirA and anti-PirB antibodies

**Figure 2:**
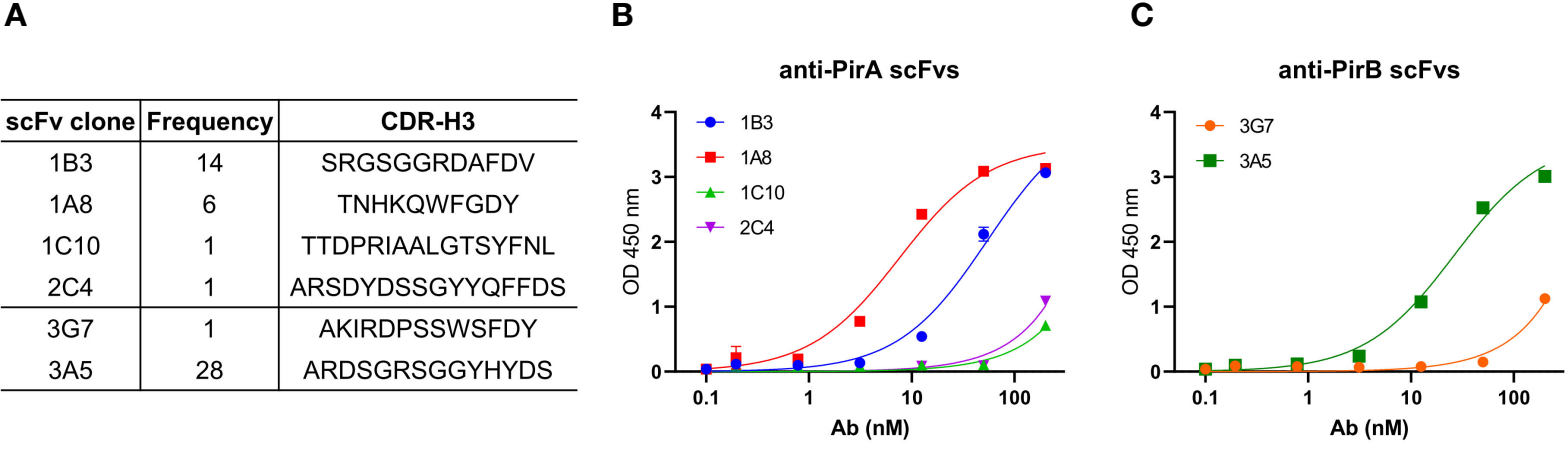
Selection of a pair of anti-PirA and anti-PirB scFvs for developing an immunoassay. **(A)** CDR-H3 sequence and frequency of anti-PirA and anti-PirB scFv clones. Four anti-PirA scFvs (1B3, 1A8, 1C10, and 2C4) and two anti-PirB scFvs (3G7 and 3A5) were identified after sequencing analysis, and the frequency of each scFv antibody was determined. **(B, C)** Validation of binding activity of scFv clones. Each scFv with 6xHis tag was purified by Ni-NTA column and was titrated to assess the binding activity to **(B)** PirA, or **(C)** PirB by ELISA.

Based on sequencing analysis of scFv antibodies, four and two scFv antibodies, which were determined at different frequencies and sequences of the third complementarity-determining region in the variable heavy chain (CDR-H3), were identified against PirA and PirB, respectively (**Figure 2A**). Each scFv expressed in the periplasmic fraction after IPTG induction was purified using a Ni-NTA column, and its binding activity to each antigen was evaluated by ELISA. As shown in **Figure 2B**, anti-PirA 1A8 scFv exhibited the highest binding activity among the tested clones, with an apparent K_D_ of 7.6 nM. In addition, 3A5 scFv bound to PirB to a greater extent than did 3G7 scFv, and the apparent K_D_ of 3A5 was estimated at 26.3 nM (**Figure 2C**). Thus, we selected 1A8 and 3A5 scFv antibodies for further use.

**Figure 3:**
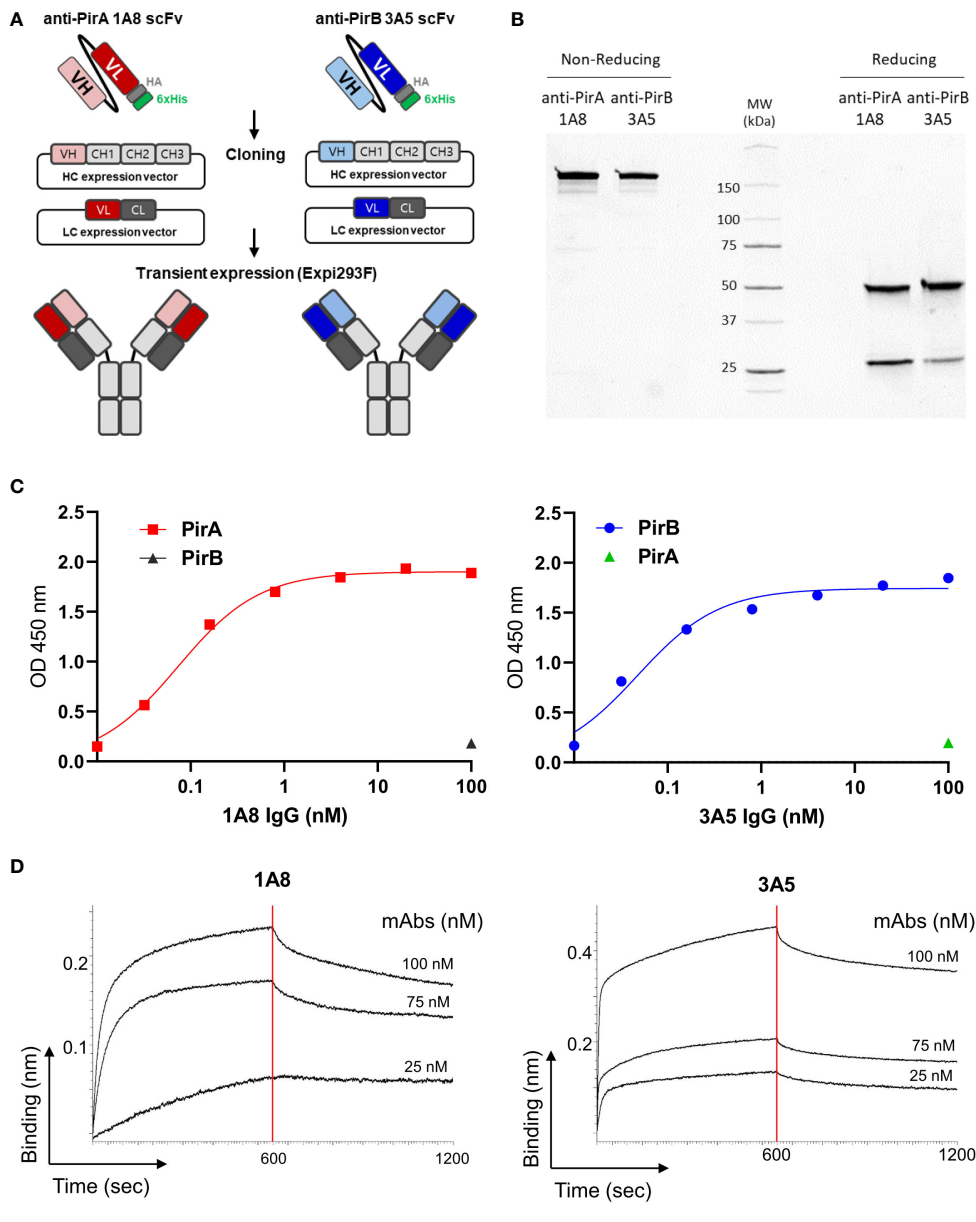
Production and validation of 1A8 and 3A5 IgGs. **(A)** Schematic illustration of transition of scFv to IgG form. VH and VL chains of the selected scFvs were separately cloned into HC and LC expression vectors containing constant regions, respectively; HC and LC expression vectors constructed for each antibody were transiently co-transfected into Expi293F cells. **(B)** Purification of 1A8 and 3A5 IgGs. IgGs expressed in supernatants were purified by protein A column and analyzed by SDS-PAGE to evaluate their intact sizes, approximately 150 kDa in non-reducing condition, and 50 kDa (HC) and 25 kDa (LC) in reduction condition. **(C)** Validation of the binding activity of 1A8 and 3A5 IgGs. Dose-dependent binding of 1A8 and 3A5 IgGs was assessed using PirA (*left*) and PirB (*right*) by ELISA, respectively. **(D)** Affinity measurement by biolayer interferometry. PirA or PirB (200 nM) was immobilized on an AR2G biosensor and allowed to bind to 1A8 (*left*) or 3A5 (*right*) IgG (25, 75, and 100 nM). Kinetic rates and equilibrium binding constants were analyzed using global fitting analysis of the binding curves.

To improve the antigen-binding activity by the avidity effect and apply it to a sandwich ELISA setting, each scFv was converted into an IgG form. As depicted in **Figure 3A**, the VH and VL regions of each scFv gene were amplified by PCR to insert two restriction sites on the N- and C-termini of the gene and cloned into the heavy chain (HC) or light chain (LC) expression vector, which was modified by inserting constant regions of the heavy or light chain. After co-transfection of HC and LC vectors constructed for each antibody into Expi293F cells, 1A8 and 3A5 were expressed, purified, and analyzed using SDS-PAGE. IgGs were verified under non-reducing and reducing conditions and showed intact and pure production (**Figure 3B**). The binding activity of the IgGs was validated by titrating each antigen. As shown in **Figure 3C**, IgGs 1A8 and 3A5 specifically bound to PirA and PirB in a dose-dependent manner, but not vice versa. We further measured the affinities (K_D_) of 1A8 and 3A5 IgG using biolayer interferometry (BLI). Recombinant PirA or PirB were immobilized on a biosensor tip, and three concentrations of each antibody were allowed to bind to each tip (**Figure 3D**). The association (K_on_) and dissociation (K_off_) values were calculated, and the K_D_ values of 1A8 and 3A5 were 1.68 nM and 0.29 nM, respectively (**Table 1**). Thus, we demonstrated that anti-PirA 1A8 and anti-PirB 3A5 are purely produced in IgG form and have a prominent binding affinity for each antigen, with promising applications in immunoassays.

### 3.3 Development of a sandwich immunoassay for the detection of heterodimeric PirAB complex

**Figure 4:**
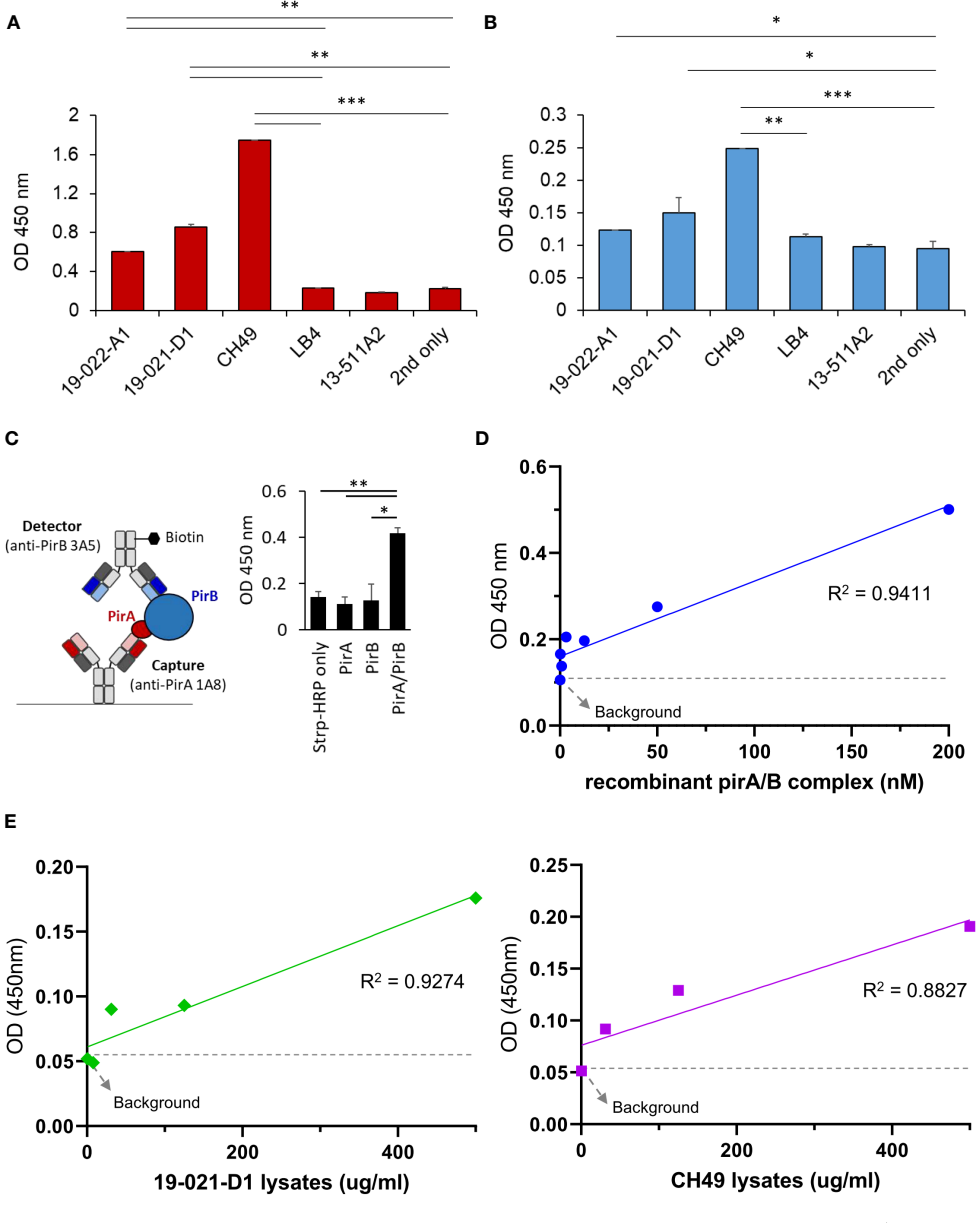
Development of a sandwich immunoassay for the detection of heterodimeric PirAB^Vp^ complex. **(A, B)** Detection of PirA and PirB in protein lysates obtained from several *Vibrio* spp. Total proteins were extracted from culture pellets of six *Vibrio* strains and coated on a 96-well plate for ELISA. Anti-PirA 1A8 and anti-PirB 3A5 IgGs were used to detect **(A)** PirA, and **(B)** PirB, separately in the lysates. Data represent the mean ± SD of duplicate tests **(A, B)**. *P < 0.05, **P < 0.01, and ***P < 0.001. **(C)** Detection of heterodimeric PirAB^Vp^ by a sandwich immunoassay. 1A8 and biotinylated 3A5 were used as a capture and a detector, respectively (*left*), and detectability of the sandwich immunoassay was assessed using PirA, PirB, and PirAB^Vp^ (*right*). Values represent the mean ± SD for a duplicate. *P < 0.05, **P < 0.01. **(D)** Sensitivity of the immunoassay in detecting PirAB. PirA and PirB were mixed together in 1:1 molar ratio, serially diluted, and detected by the immunoassay. Linear regression analysis was performed. **(E)** Detection of PirAB^Vp^ naturally produced by *Vibrio* spp. Protein lysates extracted from culture pellets of 19-021-D1 (*left*) and CH49 (*right*) were subject to the immunoassay.

Since the antibodies should detect the toxin subunits naturally expressed in *V. parahaemolyticus* strains, we investigated whether 1A8 and 3A5 exhibited binding activity towards PirA and PirB, respectively, in the protein lysates of *Vibrio* spp. To date, various AHPND-associated Vibro strains have been isolated from the environment or infected shrimp. We chose several *V. parahaemolyticus* strains in which the presence of *pirA* and/or *pirB* and the virulence causing AHPND have been confirmed (**Supplementary Table 1**). Total protein lysates were prepared from the strains, and the production of PirA or PirB was validated by ELISA using 1A8 or 3A5. As shown in **Figure 4A**, PirA was clearly detected in the lysates extracted from three strains (19-022-A1, 19-021-D1, and CH49), whereas *pirA* and *pirB* were detected by PCR, but not from *V. harveyi* (LB4) or other *V. parahaemolyticus* strains diagnosed as mutant *Vp^AHPND^* strains. 3A5 exhibited a similar pattern of PirB detection in the lysates (**Figure 4B**). Next, we designed a sandwich ELISA-based immunoassay using 1A8 IgG as a capture antibody and biotinylated 3A5 IgG as a detector for PirAB^Vp^, as depicted in **Figure 4C**. We found that the immunoassay detected not PirA or PirB but PirAB^Vp^, indicating that it could only detect the heterodimeric toxin complex and not each monomeric toxin subunit (**Figure 4C**). We also investigated the sensitivity of the immunoassay for detecting PirAB^Vp^. According to linear regression analysis after titration of recombinant heterodimeric PirAB^Vp^, the LoD of the immunoassay was found to be 201.7 ng/ml (**Figure 4D**). We further evaluated the detectability of the immunoassay for PirAB^Vp^ directly produced from *Vp^AHPND^* strains by titrating protein lysates extracted from 19-021-D1 and CH49. The linear regression analyzes showed significant coefficient of determination (R^2^) values in a concentration-dependent manner, and the detection sensitivity of the immunoassay was estimated as low as about 31.3 μg/ml of protein lysates extracted from both strains (**Figure 4E**). These data imply that the PirA and PirB toxin subunits are specifically produced by *Vp^AHPND^* strains and natively form heterodimeric PirAB^Vp^, which is detectable by the developed immunoassay.

### 3.4 Detection of PirAB^Vp^ in shrimps infected with *Vp^AHPND^* 13-028/A3

**Figure 5:**
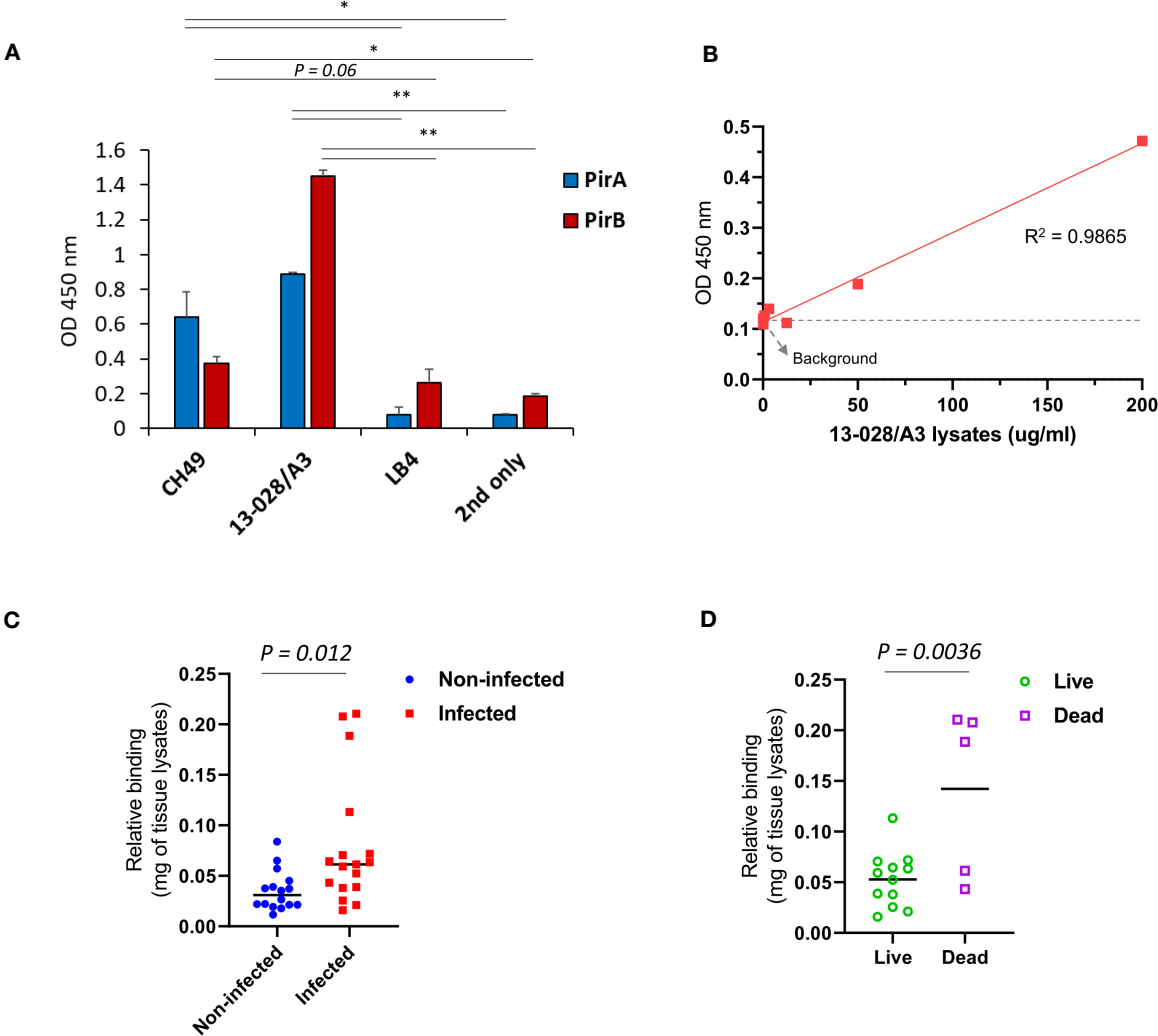
Detection of PirAB^Vp^ in shrimps infected by *V. parahaemolyticus* 13-028/A3. **(A)** Detection of the toxin subunits from the culture lysate of 13-028/A3 strain. Total proteins were extracted from the pellet of cultured bacteria and coated on an ELISA plate; the expression of PirA and PirB was determined by 1A8 and 3A5, respectively. CH49 and LB4 strains were used as a PirA/PirB-positive and -negative controls, respectively. Values represent the mean ± SD for a duplicate. *P < 0.05, **P < 0.01. **(B)** Assessment of detection sensitivity of PirAB^Vp^ in 13-028/A3 lysate. The culture lysate was serially diluted and the detection sensitivity (LoD) was determined estimated as 13.5 ug/mL. **(C, D)** Detection of PirAB^Vp^ complex in shrimps infected with 18-028/A3 strain. At day 7 after infection, hepatopancreas from non-infected or infected shrimps were dissected and lysed to prepare protein lysates. PirAB^Vp^ complex in the lysates was significantly detected in the infected group, but not in the non-infected group. The immunoassay showed a significant detection of PirAB^Vp^ complex from dead shrimps compared to live shrimps in the infected group.

We investigated whether the immunoassay could detect PirAB^Vp^ in *Vp^AHPND^*-infected shrimp. *V. parahaemolyticus* 13-028/A3, a virulent *Vp^AHPND^* strain that has been mostly used in AHPND models, was selected to infect shrimp. We first validated the expression of PirA and PirB by indirect ELISA using 1A8 and 3A5 IgGs, respectively, and determined that 13-028/A3 produced high levels of each toxin subunit (**Figure 5A**). In addition to detecting PirA and PirB, we analyzed the sensitivity in detecting PirAB^Vp^ from the lysate. As shown in **Figure 5B**, the LoD of the sandwich immunoassay was estimated to be 13.5 μg/ml by the linear regression, showing that the immunoassay could be sensitive enough to detect the heterodimeric PirAB^Vp^ from to 13-028/A3. Given the sensitivity of the immunoassay developed in this study, we examined its applicability to the diagnosis of AHPND in shrimp. We performed a challenge study by infecting shrimp with the strain and compared the PirAB^Vp^ detection activity between *Vp^AHPND^*-infected and non-infected groups. We found that the immunoassay performed using hepatopancreas lysates exhibited significant detectability of PirAB^Vp^ in the infected group compared to the non-infected group, suggesting that 13-028/A3 highly produced heterodimeric PirAB^Vp^ after infecting shrimp (**Figure 5C**). Furthermore, we assessed the amount of PirAB^Vp^ in live, moribund, and dead shrimp in the infected group. The results shown in **Figure 5D** indicate a significantly higher level of PirAB^Vp^ in HP lysates from dead shrimp than in those from live shrimp post-infection. Thus, we inferred that PirAB^Vp^ is detectable in *Vp^AHPND^*-infected shrimp using the developed immunoassay and plays a pivotal role in increasing shrimp mortality by inducing fatal virulence.

### 3.5 Structural modeling reveals the binding mode of 1A8 and 3A5 to the toxin subunits

**Figure 6:**
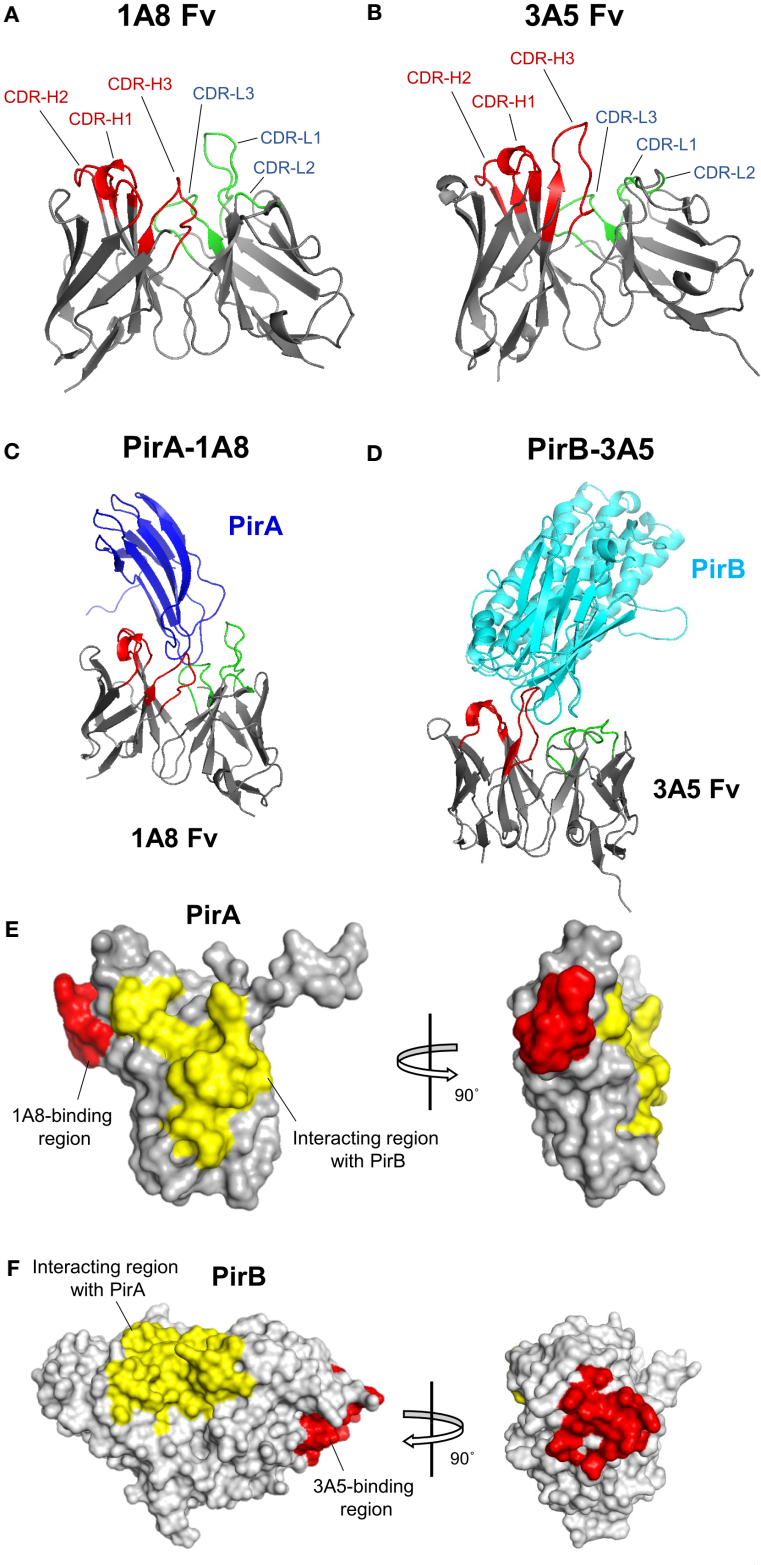
Structural docking to analyze the binding mode of 1A8 and 3A5 to each toxin subunit. **(A, B)** Antibody variable fragment (Fv) modeling. VH and VL amino acid sequences of 1A8 **(A)** or 3A5 **(B)** were used for Fv modeling using RosettaAntibody. CDR regions in VH (*red*) and VL (*green*) are highlighted in 1A8 or 3A5 Fv model. **(C, D)** Antibody-antigen docking analysis. The 1A8 Fv and PirA (PDB:3X0T) **(C)** or the 3A5 Fv and PirB (PDB:3X0U) **(D)** were utilized for structural docking analysis using ZDOCK. **(E)** Epitope analysis in 1A8-PirA docking. Potential binding sites (within 4 Å) by CDRs in VH and VL of 1A8 were analyzed based on the docking results and highlighted on PirA. Red: residues involved in 1A8 binding (W57, G58, A59, P60, F61, M62, A63, G64, G65, and K67); Yellow: residues involved in the interaction with PirB (Y11, S12, H13, D14, W15, T16, V17, V26, D27, S28, K29, H30, G104, F105, C106, T107, I108, Y109, Y110) (Lin et al., 2017). **(F)** Epitope analysis in 3A5-PirB docking. Potential binding sites (within 4 Å) by CDRs in VH and VL of 3A5 were analyzed and highlighted on PirB. Red: residues involved in 3A5 binding (I317, E318, I319, H320, Y321, N368, G369, P370, E371, Q413, E414, G415, S416, and D417); Yellow: residues involved in the interaction with PirA (Y35, A36, F37, K38, A39, M40, V41, S42, F43, G44, L45, S46, N47, M247, L248, I249, W250, Q251, K252, I253, K254, E255, L256, D260, V261, F262, V263, H264, S265, N266, L267, I268, S269, Y270, P298, N299, M300, F301, G302, E303, R304, R305, P431, D432, E433, and F434) (Lin et al., 2017).

Given the amino acid sequences of the antibodies identified in this study and the previously analyzed structural information of PirA and PirB (Lee et al., 2015), we investigated the binding site of each antibody on PirA or PirB by antibody-antigen docking analysis. Structural models of variable fragments (Fv) were generated with VH and VL sequences of the 1A8 or 3A5 antibodies using RosettaAntibody, and CDR loops in the VH (CDR-H1, -H2, and -H3) or VL (CDR-L1, -L2, and -L3) were observed in each model (**Figures 6A, B**). Using the structures of PirA and PirB, antibody-antigen docking analysis was conducted with each Fv model to determine which amino acid residues on PirA and PirB interact with the 1A8 Fv and 3A5 Fv, respectively. As shown in **Figure 6C**, the CDR-H3 and CDR-L3 loops of 1A8 seemed to interact mainly with the helical region located between the fourth and fifth beta-sheets on PirA. In the case of PirB, the docking data showed that the CDR-Hs of 3A5 were mainly involved in binding to the hydrophilic region exposed as a loop on PirB (**Figure 6D**). Given the docking analysis data, we found that 10 amino acids on PirA and 14 amino acids on PirB might be the epitope of 1A8 and 3A5, respectively, since they were the closest residues to the CDR regions (≤4 Å). Next, we visualized two regions on each antigen: (i) the antibody-binding site expected as an epitope of each antibody, and (ii) the interaction site between PirA and PirB for heterodimerization. The results shown in **Figures 6E, F** indicate that the epitopes of 1A8 and 3A5 were located away from the interaction site between PirA and PirB, respectively. Taken together, these results suggest that 1A8 and 3A5 IgGs do not interfere with the interaction site on each antigen that forms the heterodimeric PirAB^Vp^; therefore, the immunoassay was able to successfully detect recombinantly expressed and naturally produced PirAB^Vp^.

## 4 Discussion

It has been shown that PirA and PirB interact to form a heterodimeric PirAB^Vp^ and the complex causes significant mortality and morbidity in shrimp (Lee et al., 2015; Zhang et al., 2021). These results indicate that neither PirA nor PirB, but PirAB^Vp^ in binary form, is indispensable for causing significant toxicity after *Vp^AHPND^* infection in hosts. Thus, it is important to detect the binary toxin to accurately determine *Vp^AHPND^* virulence. In the present study, we demonstrated that 1A8 and 3A5 antibodies specific to PirA and PirB, respectively, can serve as a great option for the development of a novel sandwich immunoassay to detect the PirAB^Vp^ binary toxin. The immunoassay exhibited significant detectability of the binary toxin from not only the culture lysates of *Vp^AHPND^* strains, but also the protein lysates of the hepatopancreas dissected from shrimp infected with the well-known *Vp^AHPND^* strain 13-028-A3. Therefore, the novel immunoassay established in this study could specifically detect the PirAB^Vp^ binary toxin and was further developed for periodic surveillance of newly emerging *Vibrio* spp.

Although several recent studies have suggested that *V. parahaemolyticus* is the major bacterial species that can cause AHPND in shrimp because of the acquisition of a 70-kb plasmid (pVA1) encoding the binary toxin PirAB^Vp^, evidence of the transferability of pVA1-type plasmids between other *Vibrio* species have been found (Han et al., 2015; Lee et al., 2015; Dong et al., 2019). Moreover, the PirAB^Vp^ binary toxin has been identified in other *Vibrio* species belonging to the Harveyi clade, including *V. campbellii*, *V. harveyi*, and *V. owensii* (Kondo et al., 2015; Ahn et al., 2017; Dong et al., 2017; Xiao et al., 2017), and even in the Gram-positive bacterial species Micrococcus luteus (Durán-Avelar et al., 2018). PirAB^Vp^ binary toxins have also been reported in *V. punensis*, which belongs to the Orientalis clade (Restrepo et al., 2018). Whether it is still controversial that the global spread of *Vp^AHPND^* in the shrimp industry is mainly responsible for the transferability of the pVA1-type plasmids (Fu et al., 2020), the presence of *pirA* and *pirB* genes in various bacterial species definitely poses a potential risk for the spread of emerging diseases and the emergence of mutant or atypical *Vp^AHPND^* strains also acts as an internal hurdle to accurate diagnosis of the AHPND-causing *Vibrios*. Therefore, we evaluated the sensitivity of the newly developed antibody to several virulent *Vp^AHPND^* isolates, avirulent *pirA/pirB* mutant *Vp^AHPND^* isolates, and non-AHPND *Vibrio* isolates of the *Harveyi* clade. Although we were unable to confirm the potential binding activity of the newly developed antibody against the AHPND-associated virulent *Vibrio* spp. rather than *V. parahaemolyticus* because of the limited available strains, the antibody successfully detected the presence of the PirAB^Vp^ binary toxin in several virulent *Vp^AHPND^* isolates from different geographical origins, including Korea, and also clearly separated the virulent and two types of avirulent *pirA/pirB* mutant *Vp^AHPND^* isolates. Moreover, nonspecific binding of the newly developed antibody to the non-AHPND *Vibrio* isolate of the Harveyi clade was recorded. These results suggest that the newly developed antibody has strong potential for the accurate diagnosis of PirAB^Vp^ binary toxins of AHPND-causing *Vp^AHPND^* in the global shrimp industry.

The established immunoassay successfully detected natural PirAB^Vp^ in the HP of *Vp^AHPND^*-infected shrimp with an LoD of 13.5 ug/ml. Since the concentrations of hepatopancreatic proteins extracted from a single shrimp were on average 3 mg/ml (**Supplementary Table 2**), the immunoassay exhibited acceptable ability for the detection of PirAB^Vp^ in shrimp; therefore, achievement of higher sensitivity is not a significant consideration for the immunoassay. In addition, compared with the immunoassay and dot-blot methods previously reported (Wangman et al., 2017; Wangman et al., 2018), the immunoassay is the only approach available for determining the binary form of PirA and PirB subunits, indicating that the present technique is less time consuming and feasible to monitor the PirAB^Vp^ high-throughputly in a 96-well plate setting. We suggest that there is room for improvement in the immunoassay detectability. To this end, some modifications could be made by conjugating more biotin to the detector antibody 3A5 or achieving affinity maturation of capture or detector antibodies.

The crystal structures of PirA and PirB have been analyzed in previous studies, which have shown that the binary toxin is complexed heteromerically by interacting with specific amino acids in each subunit (Lee et al., 2015; Lin et al., 2017). However, the toxicity-inducing mode of action of PirAB^Vp^ remains unclear. The structural similarity between the PirAB^Vp^ binary toxin and Cry proteins has been reported, suggesting PirAB^Vp^ could work as a toxin that induces pore-forming activity in target cells (Lee et al., 2015; Lin et al., 2017). Cry proteins identified in *Bacillus thuringiensis* are insecticidal toxins composed of three active domains: i) pore-forming domain I, ii) receptor-binding domain II, and iii) sugar-binding domain III (Bravo et al., 2006; Bravo et al., 2013; Pardo-López et al., 2013; Adang et al., 2014). Based on previous investigations, the structures of the N- and C-termini of PirB are similar to those of Cry domains I and II, respectively, and the structure of PirA corresponds to Cry domain III, suggesting that the PirAB^Vp^ binary toxin possesses a Cry-like function (Lee et al., 2015). This suggests that the C-terminal PirB could bind to a receptor expressed on host cells and that the N-terminal PirB could exert pore-forming activity on the cellular membrane of the host, thereby causing cell death. Interestingly, the expected epitopes of the 3A5 antibody developed in this study were located in loops 14 (I317/E318/I319/H320/Y321), 18 (G369/P370/E371), and 22 (Q413/E414/G415/S416/D417) of PirB, corresponding to the potential receptor-binding region (**Figure 6F**). Thus, we infer that 3A5 could be utilized not only for the diagnosis of the PirAB^Vp^ binary toxin in *Vp^AHPND^*-infected shrimp, but also for further therapeutic applications based on interference with the interaction of PirB and its counterpart receptor.

## Data availability statement

The original contributions presented in the study are included in the article/**Supplementary Material**. Further inquiries can be directed to the corresponding authors.

## Ethics statement

The animal study was approved by IACUC approval no. KRIBB-AEC-21119. The study was conducted in accordance with the local legislation and institutional requirements.

## Author contributions

M-YJ: Data curation, Formal analysis, Investigation, Methodology, Validation, Writing – original draft. JEH: Formal analysis, Funding acquisition, Investigation, Methodology, Project administration, Resources, Supervision, Visualization, Writing – original draft, Writing – review & editing. DGL: Formal analysis, Investigation, Methodology, Visualization, Writing – original draft. Y-LC: Formal analysis, Investigation, Methodology, Writing – original draft. J-HJ: Investigation, Methodology, Writing – original draft. JL: Investigation, Resources, Writing – original draft. J-GP: Investigation, Resources, Writing – original draft. DHK: Investigation; Writing – review & editing. SYP: Investigation, Writing – original draft. WK: Investigation, Writing – original draft. KL: Funding acquisition, Methodology, Writing – review & editing. JHK: Conceptualization, Formal analysis, Funding acquisition, Investigation, Methodology, Project administration, Resources, Supervision, Visualization, Writing – original draft, Writing – review & editing. N-KL: Conceptualization, Formal analysis, Funding acquisition, Investigation, Methodology, Project administration, Resources, Supervision, Visualization, Writing – original draft, Writing – review & editing.
