# Supplementary material for: Novel sandwich immunoassay detects a shrimp AHPND-causing binary PirABVp toxin produced by Vibrio parahaemolyticus
Source: Front Cell Infect Microbiol. 2023 Nov 27;13:1294801. doi: 10.3389/fcimb.2023.1294801 (PMC10711049; doi:10.3389/fcimb.2023.1294801)
Supplement: Supplementary file 1 [file Table_1.docx]

Supplementary Material

# Supplementary Figures and Tables

## Supplementary Tables

**Supplementary Table 1.** *Vibrio parahaemolyticus* strains and diagnosis of pirA/pirB gene expression in them.

| **Strains** | **Originated country** | **Origin** | **Isolation date** | **pirA/pirB gene expression profile**  (Diagnosed by PCR) | **Species** | **Disease** |
| --- | --- | --- | --- | --- | --- | --- |
| 19-022-A1 | Taean, Korea | Shrimp | 2019 | *pir*A(+), *pir*B(+) | *V.parahaemolyticus* | AHPND |
| 19-021-D1 | Taean, Korea | Seawater | 2019 | *pir*A(+), *pir*B(+) | *V. parahaemolyticus* | AHPND |
| CH49 | Bangkok, Thailand | Seawater | 2019 | *pir*A(+), *pir*B(+) | *V. parahaemolyticus* | AHPND |
| 13-028/A3 | Vietnam | Shrimp | 2015 | *pir*A(+), *pir*B(+) | *V. parahaemolyticus* | AHPND |
| LB4 | USA | Seawater | 2017 | *pir*A(–), *pir*B(–) | *V.harveyi* | Non-AHPND |
| 13-511A2 | Mexico | Shrimp | 2013 | *pir*A(–), *pir*B(–) | *V. parahaemolyticus* | AHPND mutant |

Abbreviations: AHPND, acute hepatopancreatic necrosis disease.

**Supplementary Table 2.** Concentration measurement of protein lysates isolated from the hepatopancreas (HP) in a challenge study, by infecting 13-028/A3 into shrimps

| **Group** | **Sample ID** | **Status** | **HP protein lysates**  (mg/ml) |
| --- | --- | --- | --- |
| Non-infected | NC 1-2 | Live | 3.877 |
|  | NC 1-3 | Live | 4.618 |
|  | NC 1-4 | Live | 4.088 |
|  | NC 1-5 | Live | 3.174 |
|  | NC 1-6 | Live | 4.055 |
|  | NC 1-7 | Live | 5.49 |
|  | NC 2-1 | Live | 6.67 |
|  | NC 2-2 | Live | 3.438 |
|  | NC 2-3 | Live | 4.546 |
|  | NC 2-4 | Live | 4.181 |
|  | NC 2-5 | Live | 3.85 |
|  | NC 2-6 | Live | 5.314 |
|  | NC 2-7 | Live | 1.993 |
|  | NC 2-8 | Live | 3.488 |
|  | NC 2-9 | Live | 4.465 |
| 13-028/A3-infected | PC 1-1 | Dead | 0.668 |
|  | PC 1-2 | Dead | 0.854 |
|  | PC 1-3 | Dead | 2.392 |
|  | PC 1-4 | Dead | 0.713 |
|  | PC 1-5 | Live | 2.266 |
|  | PC 1-6 | Live | 1.114 |
|  | PC 1-7 | Live | 3.938 |
|  | PC 1-8 | Live | 2.973 |
|  | PC 2-1 | Dead | 1.739 |
|  | PC 2-2 | Live | 2.364 |
|  | PC 2-3 | Live | 3.651 |
|  | PC 2-4 | Live | 2.161 |
|  | PC 2-5 | Live | 1.622 |
|  | PC 2-6 | Live | 1.696 |
|  | PC 2-7 | Live | 1.495 |
|  | PC 2-8 | Live | 1.3 |
|  | PC 2-9 | Live | 3.476 |
